# DNA methylation and repressive H3K9 and H3K27 trimethylation in the promoter regions of PD-1, CTLA-4, TIM-3, LAG-3, TIGIT, and PD-L1 genes in human primary breast cancer

**DOI:** 10.1186/s13148-018-0512-1

**Published:** 2018-06-15

**Authors:** Varun Sasidharan Nair, Haytham El Salhat, Rowaida Z. Taha, Anne John, Bassam R. Ali, Eyad Elkord

**Affiliations:** 1 Cancer Research Center, Qatar Biomedical Research Institute, College of Science and Engineering, Hamad Bin Khalifa University, Qatar Foundation, Doha, Qatar; 2 Oncology Department, Al Noor Hospital, Abu Dhabi, United Arab Emirates; 3 Oncology Department, Tawam Hospital, Al Ain, United Arab Emirates; 4 Department of Pathology, College of Medicine and Health Sciences, United Arab Emirates University, Al Ain, United Arab Emirates; 5 Zayed Center for Health Sciences, United Arab Emirates University, Al Ain, United Arab Emirates; 6 Institute of Cancer Sciences, University of Manchester, Manchester, UK

**Keywords:** Breast cancer, Immune checkpoints, PD-L1, DNA methylation, Histone trimethylation

## Abstract

**Background:**

High expression of immune checkpoints in tumor microenvironment plays significant roles in inhibiting anti-tumor immunity, which is associated with poor prognosis and cancer progression. Major epigenetic modifications in both DNA and histone could be involved in upregulation of immune checkpoints in cancer.

**Methods:**

Expressions of different immune checkpoint genes and PD-L1 were assessed using qRT-PCR, and the underlying epigenetic modifications including CpG methylation and repressive histone abundance were determined using bisulfite sequencing, and histone 3 lysine 9 trimethylation (H3K9me3) and histone 3 lysine 27 trimethylation (H3K27me3) chromatin immunoprecipitation assays (ChIP), respectively.

**Results:**

We first assessed the expression level of six immune checkpoints/ligands and found that PD-1, CTLA-4, TIM-3, and LAG-3 were significantly upregulated in breast tumor tissues (TT), compared with breast normal tissues (NT). We investigated the epigenetic modifications beyond this upregulation in immune checkpoint genes. Interestingly, we found that CpG islands in the promoter regions of PD-1, CTLA-4, and TIM-3 were significantly hypomethylated in tumor compared with normal tissues. Additionally, CpG islands of PD-L1 promoter were completely demethylated (100%), LAG-3 were highly hypomethylated (80–90%), and TIGIT were poorly hypomethylated (20–30%), in both NT and TT. These demethylation findings are in accordance with the relative expression data that, out of all these genes, PD-L1 was highly expressed and completely demethylated and TIGIT was poorly expressed and hypermethylated in both NT and TT. Moreover, bindings of H3K9me3 and H3K27me3 were found to be reduced in the promoter loci of PD-1, CTLA-4, TIM-3, and LAG-3 in tumor tissues.

**Conclusion:**

Our data demonstrate that both DNA and histone modifications are involved in upregulation of PD-1, CTLA-4, TIM-3, and LAG-3 in breast tumor tissue and these epigenetic modifications could be useful as diagnostic/prognostic biomarkers and/or therapeutic targets in breast cancer.

**Electronic supplementary material:**

The online version of this article (10.1186/s13148-018-0512-1) contains supplementary material, which is available to authorized users.

## Background

Modifications in the epigenetic patterns of DNA are considered as an early event in the development of breast cancer [1, 2]. Aberrant DNA methylation and repressive histone modifications are associated with clinical and histopathological features of breast cancer such as tumor subtype, stage, and differentiation [3–5]. In mammalian cells, the DNA can be modified by the methylation of cytosine residues in CpG dinucleotides and the N-terminal histone modifications through methylation, acetylation, phosphorylation, and ubiquitination [6, 7]. The DNA methylation imprints can be erased through two different processes: (a) active demethylation occurs through demethylation enzymes such as ten-eleven translocation dioxygenase (TETs) and (b) passive demethylation by means of reduction in DNA methyl transferase (DNMTs) activity [8]. In addition to DNA demethylation, the global distribution of repressor histones such as H3K9me3 and H3K27me3 can also predominantly affect gene transcription [4].

Immune checkpoints are molecules contributing to the inhibitory pathways in the immune system and play pivotal roles in the immune evasion of tumor cells [9, 10]. Recent reports show that multiple immune checkpoint molecules are upregulated in the tumor microenvironment (TME) of breast cancer [11, 12], but the epigenetic modifications behind this upregulation are still not clear. Therefore, the epigenetic studies in breast TME could help to understand the molecular mechanisms behind their upregulation. Herein, for the first time, we investigated the epigenetic changes occurring in immune checkpoint molecules in breast TME. Initially, we found that immune checkpoints including PD-1, CTLA-4, TIM-3, and LAG-3 were upregulated in breast tumor tissues. In subsequent investigations, we examined the epigenetic modifications in tumor and normal tissues and found that the CpG motifs in the promoter regions of PD-1, CTLA-4, and TIM-3 genes were hypomethylated in tumor tissue, compared with normal tissue, but not significantly in LAG-3 promoter. Furthermore, the distribution of repressive histones including H3K9me3 and H3K27me3 was also reduced in the promoter regions of all four immune checkpoints in tumor compared with normal tissues. Collectively, our data reveal that upregulation of multiple immune checkpoints including PD-1, CTLA-4, and TIM-3 in breast TME depends on both DNA methylation and distribution of repressive histones, but the LAG-3 upregulation depends only on repressive histone distribution across their promoter region. Moreover, the relative expression of PD-L1 was the highest and TIGIT was the lowest in both NT and TT among all other immune checkpoint genes, and the demethylation percentage also agrees with the relative expression level; PD-L1 was totally demethylated and TIGIT was hypermethylated in both TT and NT.

## Results

### Upregulation of multiple immune checkpoint genes in breast tumor tissues

**Fig. 1:**
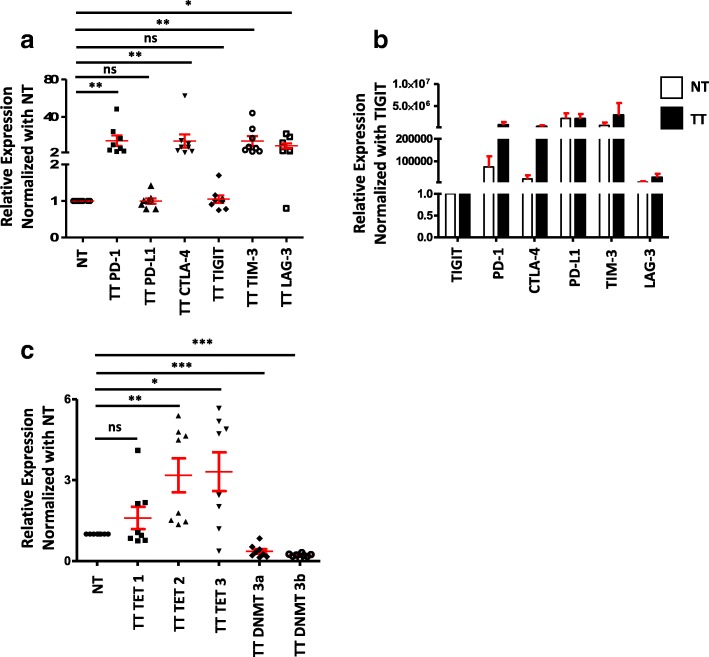
Expression of immune checkpoints and PD-L1 genes and methylation/demethylation genes in breast tumor and normal tissues. RNA isolated from breast tumor and normal tissues from eight patients were reverse transcribed to cDNA. Quantitative RT-PCR was performed to assess the expression level of PD-1, PD-L1, CTLA-4, TIGIT, TIM-3, and LAG-3 (**a**); TET1, TET2, TET3, DNMT3a, and DNMT3b (**c**) from both NT and TT. The relative expression of each gene was normalized to β-actin. Absolute expression of each gene was calculated by normalizing each gene to TIGIT expression (**b**)

Various genetic and epigenetic changes that are inherent in most of the cancer cells in TME help tumor cells to develop immune resistance mechanisms, which involves multiple immune inhibitory pathways [9]. It has been reported that in the breast TME, both malignant mammary epithelial cells and tumor-infiltrating lymphocytes (TILs) express multiple immune checkpoints/ligands, including PD-1, CTLA-4, and PD-L1 to support immune evasion [13]. To investigate the expression level of various immune checkpoint molecules in the TME, we performed quantitative real-time PCR (RT-qPCR) as described in the “Methods” section. We found that PD-1, CTLA-4, TIM-3, and LAG-3 were upregulated in tumor tissue, compared with normal tissue, while there was no change in PD-L1 and TIGIT (Fig. 1a). These data show that multiple immune checkpoint genes are upregulated in breast TME, which may help the tumor cells to evade from host anti-tumor responses. Moreover, we normalized the relative expression of all immune checkpoints with TIGIT, which is poorly expressed, and found that CTLA-4 and PD-1 were highly expressed in TT compared to NT. Interestingly, PD-L1 and TIM-3 were highly expressed in both TT and NT (Fig. 1b).

### DNA demethylation enzymes are upregulated in the breast tumor tissues

It has been reported that the loss of methylation imprints is one of the major epigenetic changes happening in human TME, compared with normal microenvironment [14]. The DNA methylation in the form of 5-methylcytosine (5mC) can be actively reversed to unmodified cytosine (5C) through ten-eleven translocation dioxygenase-mediated oxidation [15, 16]. Additionally, the methylation status can be dynamically regulated through the balance between TETs and DNMTs, and their occupancy in promoter sites can directly influence gene expression [17]. These reports prompt us to check the expression level of demethylation/methylation enzymes in breast TME. Interestingly, we found that TET2 and TET3 were significantly higher in the tumor tissues compared with normal tissues, but not TET1 (Fig. 1c). Our data are similar to the reports showing that out of all TET enzymes, TET2 and TET3 actively participate in DNA demethylation and not TET1 [18, 19]. Apart from the upregulation of TET2 and TET3, we also found that there is a significant downregulation of both DNMT3a and DNMT3b expression in cancer tissue compared with normal tissues (Fig. 1c). These data reveal that the epigenetic modifications such as DNA demethylation may play a predominant role in the upregulation of immune checkpoints in breast TME.

### The promoter regions of PD-1, CTLA-4, and TIM-3 are significantly hypomethylated in tumor, compared with normal tissues

**Fig. 2:**
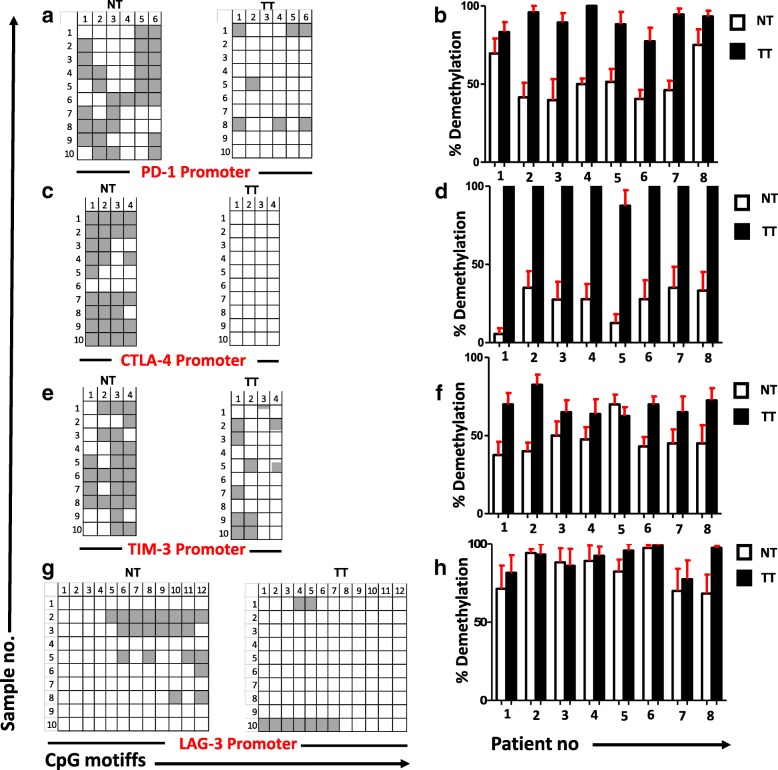
Analyses of CpG methylation status of immune checkpoint promoters in breast tumor and normal tissues. CpG methylation status of the promoter regions of PD-1 (**a**), CTLA-4 (**c**), TIM-3 (**e**), and LAG-3 (**g**) were analyzed by bisulfite sequencing of the genomic DNA isolated from breast tumor and normal tissues from eight patients. Representative plots from eight individual tumor and normal tissues show the methylation status of CpG motifs. Methylation status of individual CpG motifs is shown by white (demethylation) or gray (methylation) colors. The bar charts show the demethylation percentage of PD-1 (**b**), CTLA-4 (**d**), TIM-3 (**f**), and LAG-3 (**h**) from eight different NT and TT samples

**Fig. 3:**
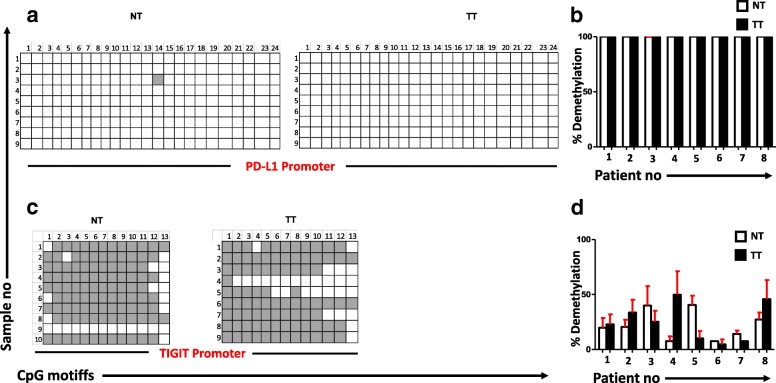
Analyses of CpG methylation status of PD-L1 and TIGIT promoters. CpG methylation status of the promoter regions of PD-L1 (**a**) and TIGIT (**c**) were analyzed by bisulfite sequencing of the genomic DNA isolated from the breast tumor and normal tissues. Representative plots from eight individual tumor and normal tissues show the methylation status of CpG motifs. Methylation status of individual CpG motifs is shown by white (demethylation) or gray (methylation). The bar charts show the demethylation percentage of PD-L1 (**b**) and TIGIT (**d**) from eight different NT and TT samples

**Fig. 4:**
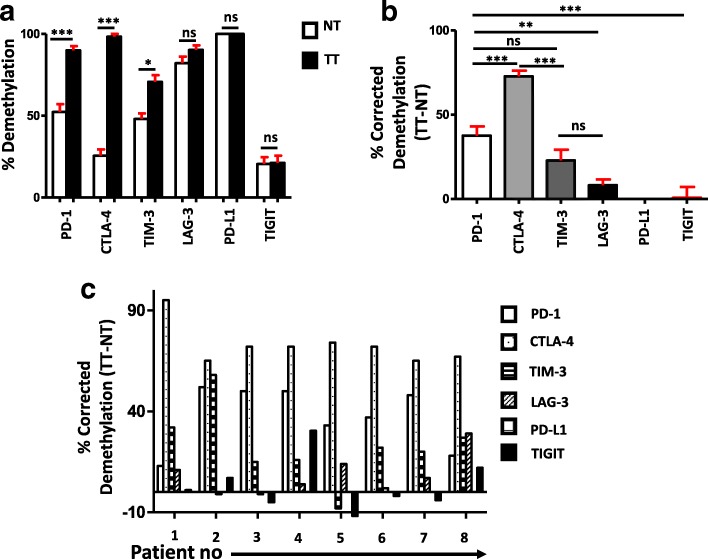
Corrected demethylation percentage of immune checkpoint promoters in tumor tissues. CpG methylation status of the promoter regions of PD-1, CTLA-4, TIM-3, LAG-3, PD-L1, and TIGIT was analyzed by bisulfite sequencing of the genomic DNA isolated from breast and normal tissues from eight patients. A bar diagram shows the average demethylation percentage from the eight NT and TT samples of each gene (**a**). A bar diagram shows the corrected demethylation percentage of immune checkpoints by subtracting average demethylation percentage of NT from TT (**b**). A bar diagram shows the corrected demethylation percentage of all six genes in individual patients (**c**)

To check the DNA epigenetic modifications in immune checkpoints/ligand in the breast TME, we examined the promoter CpG methylation profile of genes including PD-1, CTLA-4, TIM-3, LAG-3, PD-L1, and TIGIT in tumor and normal tissues. It has been reported that the CpG islands (CGIs), especially those within the promoter regions, play a vital role in tumorigenesis, genome imprinting, gene silencing, and X-chromosome inactivation [20]. Due to this multifunctional importance of CGIs in transcription regulation and epigenetic modification, we intended to check the promoter CpG methylation pattern of PD-1, CTLA-4, TIM-3, LAG-3, PD-L1, and TIGIT. Herein, we selected 6 CpGs from PD-1, 4 CpGs from CTLA-4, 4 CpGs from TIM-3, 12 CpGs from LAG-3, 24 CpGs from PD-L1, and 13 CpGs from TIGIT promoters to determine the methylation profiles (Figs. 2a, c, e and g, 3a and c). Additionally, while comparing the methylation status of tumor tissues between the eight patients, DNA demethylation percentage of CTLA-4 in TT looks more consistent than the other five genes (Figs. 2b, d, f and h, 3b and d). Interestingly, we found that the CpGs of PD-L1 promoter was completely demethylated and TIGIT was prominently methylated with no significant differences between NT and TT (Fig. 3). The average demethylation percentage of PD-1 promoter among the eight patients looks similar to CTLA-4; TT was more demethylated (90%) than NT (54%) (Fig. 4a). Furthermore, TIM-3 promoter also appears more demethylated in TT (69%) than NT (38%) (Fig. 4a). There were no significant changes in demethylation status of LAG-3 promoter in TT (91%) compared to NT (83%) (Fig. 4a). Therefore, we concluded that in breast TME, the expressions of PD-1, CTLA-4, and TIM-3 are epigenetically regulated through DNA methylation and for the LAG-3 upregulation; other epigenetic modifications might be involved. The demethylation percentage was highest in PD-L1 and lowest in TIGIT with no significant differences between TT and NT (Fig. 4a). These data correlate with the data in Fig. 1b that there is no significant difference between the relative expression of PD-L1 and TIGIT in TT and NT. Next, we wanted to see the actual demethylation percentage in TT samples by excluding the NT demethylation percentage. In accordance with our previous results, we found that CTLA-4 is more demethylated followed by PD-1, TIM-3, LAG-3, and finally PD-L1 and TIGIT (Fig. 4b). Additionally, we also checked the actual demethylation percentage in PD-1, CTLA-4, TIM-3, LAG-3, PD-L1, and TIGIT genes in individual patient samples and found that in TT samples, the demethylation percentage of CTLA-4 is consistently higher in all patients compared with all other five genes (Fig. 3c). Taken together, our data show that DNA demethylation plays a vital role in the upregulation of CTLA-4, PD-1, and TIM-3 in breast TME. Moreover, the expression of CTLA-4 could be strongly regulated by DNA methylation.

### Repressive histone bindings to promotors of PD-1, CTLA-4, TIM-3, and LAG-3 are significantly reduced in breast tumor tissues

**Fig. 5:**
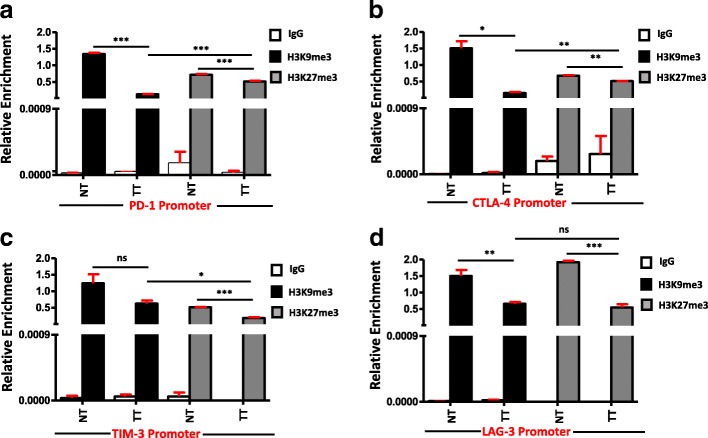
Analyses of distribution of H3K9me3 and H3K27me3 across the promoters of immune checkpoints in tumor and normal tissues. Cells from two individual NT and TT samples were isolated by enzyme disaggregation. Chromatin was precipitated using H3K9me3, H3K27me3 antibodies, and control IgG as negative control. Subsequent qPCR was performed using PD-1, CTLA-4, TIM-3, and LAG-3 promoter primers and data were normalized to input. ChIP analysis of distribution of H3K9me3 and H3K27me3 at PD-1 (**a**), CTLA-4 (**b**), TIM-3 (**c**), and LAG-3 (**d**) promoter regions are shown

The regulation of gene expression is not limited to DNA methylation, but also depends on histone posttranslational modifications (HPTMs). It has been reported that DNA methylation can elicit the changes in chromatin structure including histone deacetylation, methylation, and local chromatin compaction [21]. H3K9me3 and H3K27me3 are two known histone marks that seem to promote chromatin compaction in promoter regions, which could be associated with hindrance of transcriptional activation [22, 23]. To quantify the binding intensity of H3K9me3 and H3K27me3 on the promoters of PD-1, CTLA-4, TIM-3, and LAG-3 in normal and breast tumor tissues, we performed a ChIP-qPCR analysis using H3K9me3 and H3K27me3 antibodies. We found that in PD-1 and CTLA-4 promoters, both repressive histones bind weakly in tumor tissues compared with normal tissues (Fig. 5a, b). Additionally, in both PD-1 and CTLA-4 promoters, H3K9me3 binds significantly weaker than H3K27me3 in tumor tissues (Fig. 5a, b). Moreover, in TIM-3 promoter, only H3K27me3 binds weakly in tumor tissues and there is no significant difference in the binding of H3K9me3 in tumor compared to normal tissues (Fig. 5c). In case of LAG-3 promoter, both repressive histones bind weakly in tumor tissues compared to normal tissues (Fig. 5d). Collectively, our data show that the regulation of PD-1, CTLA-4, and LAG-3 were under the control of H3K9me3 and H3K27me3, but TIM-3 is regulated only by H3K27me3. In breast TME, these repressive histones fail to bind with the specific promoters of PD-1, CTLA-4, TIM-3, and LAG-3, which could lead to their upregulation of gene expression.

## Discussion

Expression of immune checkpoint molecules on T cells is identified as one of the important regulatory mechanisms of immune cells to regulate responses against self-antigens [10, 24]. Reports show that individual or multiple immune checkpoints are expressed on immune cells, which can functionally synergize each other in various disease contexts [25, 26]. Even though the precise signaling pathways behind the expression of these checkpoints are poorly understood, pre-clinical studies conducting with blockade of multiple checkpoints suggest that each of these molecules follows relatively unique pathways [27, 28]. Recent advances in breast cancer research have emphasized that epigenetic modifications are correlated with cancer development and progression [5, 29, 30]. An improved understanding of these epigenetic regulations can pave the way to advance the concept of targeting immune checkpoints in cancer immunotherapy. This study critically advances our knowledge of the epigenetic modifications behind the transcriptional upregulation of PD-1, CTLA-4, TIM-3, LAG-3, TIGIT, and PD-L1 in breast tumor tissues.

In breast cancer, it has been reported that multiple immune checkpoint molecules were upregulated in TILs [12, 31–33], and their expression correlates with tumor progression [13]. In our study, we checked the expression of immune checkpoints/ligand including PD-1, CTLA-4, TIGIT, TIM-3, LAG-3, and PD-L1 and found that PD-1, CTLA-4, TIM-3, and LAG-3 were upregulated in breast tumor tissues, compared with normal tissues (Fig. 1a). To investigate the molecular mechanism behind this upregulation, we checked the epigenetic modifications in these genes. Previous reports revealed that breast carcinogenesis is in fact a multistep process, which involves both genetic and epigenetic modifications [5, 29, 34], leading to genetic abnormalities in oncogenes and/or tumor suppressor genes [35–37]. These reports prompt us to check the expression of demethylation enzymes (TETs) and methylation enzymes (DNMTs) in tumor and normal tissues and found that TET2 and TET3 were upregulated, while DNMT3a and DNMT3b were downregulated in the TME (Fig. 1c). Our data are similar to previous findings that TET proteins are predominantly expressed in breast tissue [38] and play a key role in regulating hypoxia-enhanced tumor malignancy [39]. Some recent reports showed that DNMT inhibitors (DNMTi), such as 5-azacytidine and 5-decitabine, can hypomethylate the promoter region of PD-1, PD-L2, and CTLA-4 in patients with acute myeloid leukemia with myelodysplastic syndromes (AML/MDS) and upregulate their expression [40–42]. These results are in line with our findings that the downregulation of DNMT3a and 3b can significantly hypomethylate the promoter regions of PD-1, CTLA-4, and TIM-3, which in turn upregulate their expression levels in the TME (Figs. 1 and 2). Moreover, there were no changes in the methylation status of LAG-3, PD-L1, and TIGIT, indicating that genes follow different methylation patterns in the breast TME.

Various epigenetic alterations including methylation of CpG motifs in promoter regions, changes in chromatin structure, and binding of histone complexes to promoter regions can ultimately lead to the activation of oncogenes by silencing tumor suppressor genes [43]. The transcriptomic and methylation profiles of CTLA-4 and PD-1 in non-small cell lung cancer (NSCLC) patients showed that hypomethylation in the CpG islands of these genes was strongly correlated with their increased expression in the TME compared with normal tissue [44]. To reveal the underlying mechanism behind the upregulation of multiple immune checkpoints in breast tumor, we selected certain CpGs from the promoter regions of PD-1, CTLA-4, TIM-3, LAG-3, PD-L1, and TIGIT and investigated their methylation status in tumor tissues and control non-tumor tissues from all eight patients. Our results show that tumor environment alters the promoter methylation profile of PD-1, CTLA-4, and TIM-3 to extremely hypomethylated state. It has been reported that gene transcription is tightly correlated with the CpG methylation profile, where they are activated by hypomethylation and silenced by hypermethylation [45]. The promoter CpG methylation profile shows that this hypomethylation could be the reason behind the upregulation of checkpoints in breast tumor tissues (Fig. 2a, c and e). Furthermore, while comparing the six genes, CpG regions of CTLA-4 in tumor tissue were totally demethylated, which articulates that this demethylation could be a key reason behind its upregulation in breast TME. We also checked the percentage of demethylation across the eight patients and found that in PD-1 and CTLA-4, the percentage was increased consistently in tumor tissue of all patients but not in TIM-3. These data show that the consistent demethylation status of PD-1 and CTLA-4 promoters in tumor tissue could be utilized as a diagnostic marker for breast cancer. A recent study showed that PD-L1 promoter was hypomethylated in tumors with dense T cell infiltration, which correlated with adverse prognosis, and anti-PD-1 therapy could reverse this hypomethylation status [46]. Another study showed that the PD-1, CTLA-4, and PD-L1 promoter hypomethylation has a significant impact on the course of NSCLC progression [44]. These reports and our study rationalize the use of DNA methylation as a prognostic biomarker in cancer. The methylation profile analysis of PD-L1 and TIGIT showed that PD-L1 was completely demethylated and TIGIT was prominently methylated in both TT and NT (Fig. 3). This could be the reason that there were no significant changes in the relative expression between NT and TT for these two genes. Moreover, in LAG-3, we did not find a significant difference in demethylation percentage between tumor and normal tissues. This can be explained as the upregulation of LAG-3 in tumor tissue is not dependent on the DNA methylation but could be regulated by some other epigenetic modifications.

Apart from DNA methylation, post-translational histone modifications are also involved in the regulation of gene expression in breast cancer pathogenesis and its diversity [47]. It has been reported that the establishment of basic DNA methylation profile is mediated through histone modifications by recruiting DNMTs to the target CpGs [48]. To check the association of histone methyl marks in the promoter region of immune checkpoints, we selected the two known repressive histones, H3K9me3 and H3K27me3, that act to impede transcriptional elongation thereby silencing genes [49]. We found that the distribution of both trimethyl histones in the promoter region of PD-1, CTLA-4, and LAG-3 was reduced in tumor compared with normal tissues. Furthermore, only the distribution of H3K27me3 was reduced in the tumor tissue of TIM-3 promoter. These results show that the epigenetic modification behind the upregulation of immune checkpoints was not limited to DNA methylation but also depends on the distribution of the methylated histones across promoters.

Immune checkpoint inhibition is considered as one of the recent successful therapeutic modalities. Despite the clinical success, one of the main limitations in immune checkpoint therapy is the low response rate among patients [50]. The dynamic and reversible nature of epigenetic modifications occurring in certain gene loci in the TME makes them a relevant target for cancer therapy [51]. Apart from DNA and histone methylation, other modifications such as histone acetylation and expression of microRNAs (miRNA) and long noncoding RNAs (lncRNAs) can also regulate immune checkpoints in the TME. Interestingly, preclinical studies showed that the histone deacetylase inhibitors (HDACi) can augment the response of PD-1 immunotherapy in lung adenocarcinoma and melanoma [52, 53]. Moreover, other reports showed that the expression of miRNAs including miR-330-5p, mir-138, and miR-424(322) regulates the expression of TIM-3, CTLA-4, and PD-1/PD-L1 in AML [52], Glioma [54], and ovarian carcinomas [55], respectively. Of note, the current scenario of epigenetic therapy is mainly focused on DNMTi and HDACi [56]. In a preclinical NSCLC study, it was shown that mocetinostat, a class I/IV HDACi, together with anti-PD-L1 antibody augmented the antitumor activity by decreasing the immune suppressive cell types and increasing CD8^+^ T cell infiltration in the TME [57]. Some recent reports showed that in breast cancer models, HDAC inhibition together with immune checkpoint blockades can reduce tumor growth and metastasis [58, 59]. In order to improve the cancer immunotherapeutic arsenal, novel combination therapies should be established. Recent advances in research support combination of epigenetic modulators with immune checkpoint inhibitors as a more efficient approach for cancer therapy [60–62]. Our data advance the current knowledge on the importance of epigenetic modifications for the upregulation of immune checkpoints and their ligands in the breast TME.

## Conclusions

**Fig. 6:**
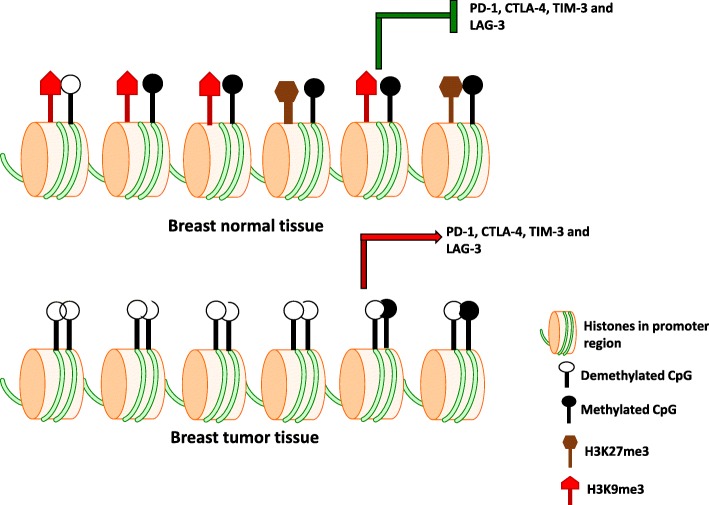
Schematic diagram shows the transcriptional regulation of immune checkpoints by epigenetic modifications in breast tumor microenvironment. The transcriptional upregulation of PD-1, CTLA-4, TIM-3, and LAG-3 in breast tumor microenvironment are dependent on both DNA methylation and the distribution of H3K9me3 and H3K27me3 across their promoter regions. PD-L1 and TIGIT are not included in this schematic because there is no difference in their methylation profile between tumor and non-tumor microenvironment, as described in the text

In conclusion, our study shows that multiple immune checkpoints including PD-1, CTLA-4, TIM-3, and LAG-3 are upregulated in breast tumor tissues. The epigenetic modifications behind their upregulations are dependent on DNA methylation and the repressive histone distribution. Furthermore, upregulation of LAG-3 depends only upon repressive histone distribution. The overall conclusion is graphically represented in Fig. 6.

## Methods

### Sample collection

Tumor tissues (TT) and adjacent non-cancerous normal tissues (NT) were obtained from eight breast cancer patients who underwent surgery at Tawam Hospital, Al Ain. All patients included in the study were treatment-naive prior to surgery. Table 1 shows the clinical and pathological characteristics of all participating subjects. The study was executed under the ethical approval by Al Ain Medical District Research Ethics committee, Al Ain, United Arab Emirates (Protocol no. 13/23-CRD 244/13). All patients provided written informed consent prior to sample collection. All experiments were performed in accordance with the relevant guidelines and regulations. After sample collection, the tissues were stored in liquid nitrogen with freezing media.

**Table 1:** Characteristic features of study population

S No	Age	ER	PR	HER2	Grade	Histological grade	TNM stage
1	43	+	+	−	I	NA	IA
2	47	−	−	+	II	Moderate	IA
3	59	+	+	+	III	Poorly differentiated	IIB
4	43	−	−	+	III	Poorly differentiated	IIB
5	65	+	−	−	II	Moderate	IIB
6	41	−	−	−	III	Poorly differentiated	IIA
7	54	+	+	−	III	Poorly differentiated	IA
8	43	+	+	NA	I	Well differentiated	IA

*NA* not available

### DNA and RNA isolation

DNA and RNA were isolated using RNA/DNA/Protein Purification Plus Kit (Norgen Biotek Corp, Ontario, Canada) as per manufacturer’s instructions from eight TT and their corresponding NT. Briefly, frozen tissues were transferred into a mortar containing adequate amount of liquid nitrogen and grind the tissue thoroughly using a pestle followed by resuspending with lysis buffer and collection in an Eppendorf tube. The tubes were incubated at 55 °C for 10 min followed by DNA extraction using DNA extraction column. The flow-through from DNA extraction was used for RNA and protein extractions. After DNA extraction, the RNA was extracted using RNA extraction column and the flow-through was used for protein extraction. The DNA and RNA concentrations were measured using NanoDrop 2000c (Thermo scientific, Massachusetts, USA) and stored at − 80 °C.

### Quantitative real-time PCR

One microgram of RNA from each sample was reverse transcribed into cDNA using QuantiTect Reverse Transcription Kit (Qiagen, Hilden, Germany). PCR reactions were performed on QuantStudio 7 Flex qPCR (Applied Biosystems, California, USA) using Fast SYBER Green Master Mix (Applied Biosystems). All data were normalized to β-actin. Non-specific amplifications were checked by the use of melting curve and agarose gel electrophoresis. The relative changes in target gene expression were analyzed by using 2-^ΔΔCT^ method. The absolute expression of immune checkpoints in both TT and NT was checked by comparing the relative expression values of all checkpoints normalized to the relative expression values of TIGIT. Sequences of primers are listed in Additional file 1: Table S1a. The primers were designed using Primer3 (http://www.ncbi.nlm.nih.gov/tools/primer-blast/).

### CpG methylation analysis by bisulfite sequencing

The genomic DNA was extracted from tumor and normal tissues as described before and treated with bisulfite using the EZ DNA Methylation Kit (Zymo Research, Irvine, CA, United States). The bisulfite-treated DNA was then subjected to PCR for the amplification of the promoter regions of PD-1, CTLA-4, TIM-3, and LAG-3 using Hot Start TaKaRa Taq DNA Polymerase (TaKaRa Bio, Shiga, Japan). PCR primers were designed using MethPrimer software (http://www.urogene.org/methprimer/index1.html), all are listed in Additional file 1: Table S1b. The PCR products obtained were cloned into the pGemT-easy vector (Promega, Madison, USA) using DNA Ligation Kit, Mighty Mix (TaKaRa Bio). Ten individual clones from each sample were purified using Wizard® Plus SV Minipreps DNA Purification System (Promega) and sequenced with M13-reverse/forward primers (Additional file 1: Table S1c). The promoter sequence details of immune checkpoints and PD-L1 genes are shown in Additional file 2: Figure S2 A-F.

### Enzyme disaggregation of tumor and normal tissues for cell isolation

Enzyme disaggregation (ED) of frozen tumor and normal tissues from two breast cancer patients was performed on a roller mixer at 37 °C for 60 min. Briefly, after thawing the vials, tissues were first washed with phosphate buffer saline (PBS) and mechanically cut into small fragments (2–4 mm) using a surgical scalpel. Tissues were then suspended into RPMI-1640 with 1% penicillin/streptomycin and enzyme cocktail consisting of 1 mg/ml collagenase, 100 μg/ml hyluronidase type V, and 30 IU/ml of deoxyribonuclease I (all from Sigma-Aldrich, UK). Cell suspension was then passed through a 100-μm BD Falcon cell strainer (BD Biosciences, Oxford, UK) to remove debris and aggregates. Cells were then washed with serum-free RPMI-1640 and resuspended in RPMI-1640 enriched with 10% FCS and 1% penicillin/streptomycin and used for chromatin immunoprecipitation (ChIP) experiments.

### Chromatin immunoprecipitation assay

Cells isolated from NT and TT through ED were subjected to ChIP analysis using Zymo-Spin ChIP kit (Zymo Research) as per manufacturer’s instructions. Briefly, after cell isolation by ED, nuclear lysate was prepared as per the protocol and sonicated using Omni Sonic Ruptor 400 Ultrasonic Homogenizer (OMNI International, GA, USA) to make small DNA fragments ranging from 100 to 1000 base pairs and then incubated with ChIP grade anti-Histone H3 (tri methyl K9) rabbit mAb (Abcam, Cambridge, United Kingdom) and anti-Histone H3 (tri methyl K27) rabbit mAb (Abcam). Isotype-matched control Ab was used as negative control. Immune complexes containing DNA fragments were precipitated using Magna A/G beads supplied with the kit. Relative enrichment of the target regions in the precipitated DNA fragments was analyzed by QuantStudio 7 Flex qPCR (Applied Biosystems) using Fast SYBER Green Master Mix (Applied Biosystems). All data were normalized to input controls. Non-specific amplification was checked by the use of melting curve and agarose gel electrophoresis. Sequences of primers are listed in Additional file 1: Table S1d.

### Sanger sequencing

The purified plasmid DNA samples were subjected to sequencing using 3130X Genetic Analyzer (Applied Biosystems). Briefly, the cycle sequencing reactions of samples were performed using M-13 forward/reverse primers and BigDye Treminator V3.1 (Applied Biosystems). Thermocycler conditions were as follows: 95 °C for 5 min, 35 cycles of 95 °C for 30 s, and 60 °C for 4 min. After PCR reaction, DNA was precipitated using 125 mM EDTA and 95% ethanol by incubation at − 20 °C for 30 min. After incubation, DNA was washed twice with 70% ethanol followed by denaturation using formaldehyde. Denatured DNA was then loaded into analyzer for sequencing.

### Statistical analyses

Statistical analyses were performed using GraphPad Prism 5 software (GraphPad Software, USA). We checked normality using Shapiro-Wilk normality test. Paired *t* test was done for the samples passed normality test, and for others, nonparametric/Wilcoxon matched-pairs signed rank test was performed. The *P* values are represented as follows: ***P < 0.001, **P < 0.01, and *P < 0.05. A *P* value of > 0.05 is considered statistically non-significant (NS). The data are presented as mean + standard error of the mean (SEM).

## Additional files

Additional file 1: **Table S1.** Primer sequences used in this study. (DOCX 18 kb)

Additional file 2: **Figure S1.** Methylation PCR and promoter sequences of immune checkpoints and PD-L1 genes. The upper representative blots show gel electrophoresis of the PCR products from NT or TT after bisulfite treatment using methyl primers. Lower figures show the promoter sequences with the primer details (red) and CpG sites (blue) for PD-1 **(A)**, CTLA-4 **(B)**, TIM-3 **(C)**, LAG-3 **(D)**, PD-L1 **(E)**, and TIGIT **(F)**. (PPTX 2479 kb)

## Abbreviations

ChIP: Chromatin immunoprecipitation
CpGI: CpG islands
CTLA-4: Cytotoxic T-lymphocyte associated protein 4
DNMT: DNA methyltransferase
DNMTi: DNA methyltransferase inhibitor
H3K27me3: Histone 27 lysine 9 trimethylation
H3K9me3: Histone 3 lysine 9 trimethylation
HDACi: Histone deacetylase inhibitor
LAG-3: Lymphocyte-activation gene 3
PD-1: Programmed cell death protein-1
PD-L1: Programmed death-ligand 1
RT-qPCR: Quantitative real-time PCR
TET: Ten-eleven translocation dioxygenase
TIGIT: T cell immunoreceptor with Ig and ITIM domains
TIM-3: T-cell immunoglobulin and mucin-domain containing-3
