# Supplementary material for: DNA methylation and repressive H3K9 and H3K27 trimethylation in the promoter regions of PD-1, CTLA-4, TIM-3, LAG-3, TIGIT, and PD-L1 genes in human primary breast cancer
Source: Clin Epigenetics. 2018 Jun 15;10:78. doi: 10.1186/s13148-018-0512-1 (PMC6003083; doi:10.1186/s13148-018-0512-1)
Supplement: Supplementary file 1 — Table S1. Primer sequences used in this study. (DOCX 18 kb) [file 13148_2018_512_MOESM1_ESM.docx]

**Additional file 1**

**Table S1: Primer sequences used in this study**

1. **RT-qPCR primers**

| **Genes** | **Forward primer (5′-3′)** | **Reverse primer (5′-3′)** |
| --- | --- | --- |
| PD-1 | CCAGGATGGTTCTTAGACTCCC | TTTAGCACGAAGCTCTCCGAT |
| PD-L1 | TGGCATTTGCTGAACGCATTT | TGCAGCCAGGTCTAATTGTTTT |
| CTLA-4 | GCCCTGCACTCTCCTGTTTTT | GGTTGCCGCACAGACTTCA |
| TIGIT | TCTGCATCTATCACACCTACCC | CCACCACGATGACTGCTGT |
| TIM-3 | TCCAAGGATGCTTACCACCAG | GCCAATGTGGATATTTGTGTTAGATT |
| LAG-3 | GCGGGGACTTCTCGCTATG | GGCTCTGAGAGATCCTGGGG |
| TET-1 | CATCAGTCAAGACTTTAAGCCCT | CGGGTGGTTTAGGTTCTGTTT |
| TET-2 | GATAGAACCAACCATGTTGAGGG | TGGAGCTTTGTAGCCAGAGGT |
| TET-3 | GCCGGTCAATGGTGCTAGAG | CGGTTGAAGGTTTCATAGAGCC |
| DNMT3a | CCGATGCTGGGGACAAGAAT | CCCGTCATCCACCAAGACAC |
| DNMT3b | AGGGAAGACTCGATCCTCGTC | GTGTGTAGCTTAGCAGACTGG |
| β-ACTIN | AGAGCTACGAGCTGCCTGAC | AGCACTGTGTTGGCGTACAG |

1. **PCR primers for CpG methylation analysis**

| **Genes** | **Forward primer (5′-3′)** | **Reverse primer (5′-3′)** |
| --- | --- | --- |
| PD-1 promoter | GGGAAGGTAGAGGTTAGGTATTTGT | ACCCCTTCCTCCTCTATATCTCTACT |
| CTLA-4 promoter | TTATGGATTGGTTTGTTTTGTTTAGT | CAAAACCTTTCAAAATCCTAAAACTT |
| TIM-3 promoter | AGGTTGGAGTGTAATGGTATGATTT | TCAAAAACAACCTAACCAACATAAA |
| LAG-3 promoter | GGTGTTTTTGTATGTTTGAAATTTAG | AACAACCCTCTAACCTCATTTACTC |
| PD-L1 promoter | TTGTTTTGGGTAGAGGTGGG | AAAAACCAAATACATTACCTATTCTTA |
| TIGIT promoter | GGTTATATAAAGGGTTTGGT | TAAATAACTACACTAATAAACAAAACAAAT |

**c) Sequencing Primer**

| **Genes** | **Forward primer (5′-3′)** | **Reverse primer (5′-3′)** |
| --- | --- | --- |
| M-13 | TGTAAAACGACGGCCAGT | CAGGAAACAGCTATGACC |

1. **ChIP-qPCR primers**

| **Genes** | **Forward primer (5′-3′)** | **Reverse primer (5′-3′)** |
| --- | --- | --- |
| PD-1 promoter | CCTCACATCTCTGAGACCCG | CCGAAGCGAGGCTAGAAACC |
| CTLA-4 promoter | GAGGACCCTTGTACTCCAGGAA | CGAAAAGACAACCTCAAGCACTC |
| TIM-3 promoter | TGTTGGTCAGGCTGTTCTTG | AGGGGGCTTATGCTCTCATT |
| LAG-3 promoter | TCCAGTCAGTGCGACAAAAG | GCATCCCTGAGAATCCAAAA |
