# Supplementary material for: DNA methylation and repressive H3K9 and H3K27 trimethylation in the promoter regions of PD-1, CTLA-4, TIM-3, LAG-3, TIGIT, and PD-L1 genes in human primary breast cancer
Source: Clin Epigenetics. 2018 Jun 15;10:78. doi: 10.1186/s13148-018-0512-1 (PMC6003083; doi:10.1186/s13148-018-0512-1)
Supplement: Supplementary file 2 — Figure S1. Methylation PCR and promoter sequences of immune checkpoints and PD-L1 genes. The upper representative blots show gel electrophoresis of the PCR products from NT or TT after bisulfite treatment using methyl primers. Lower figures show the promoter sequences with the primer details (red) and CpG sites (blue) for PD-1 (A), CTLA-4 (B), TIM-3 (C), LAG-3 (D), PD-L1 (E), and TIGIT (F). (PPTX 2479 kb) [file 13148_2018_512_MOESM2_ESM.pptx]

## Slide 1
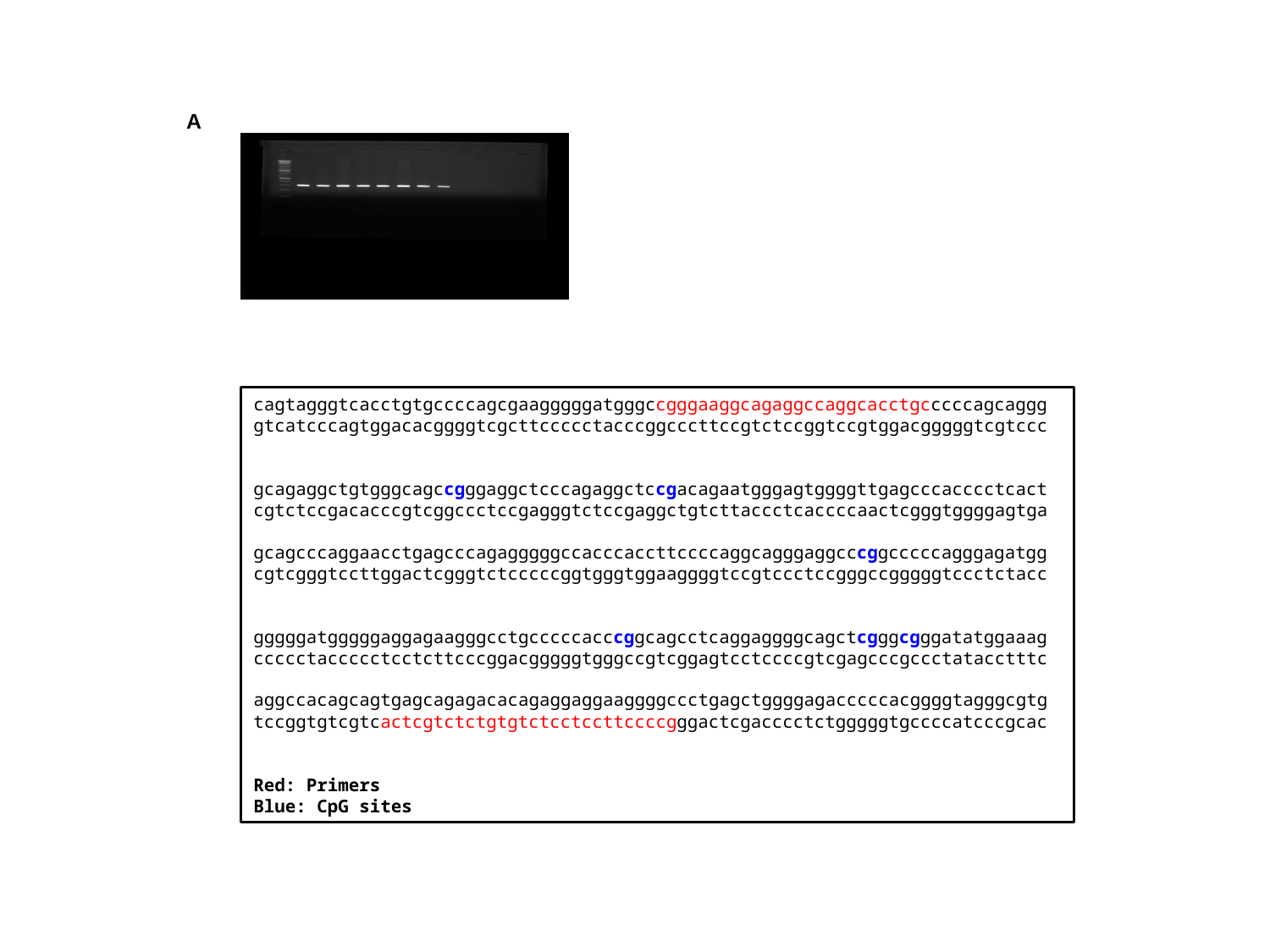

A
cagtagggtcacctgtgccccagcgaagggggatgggccgggaaggcagaggccaggcacctgcccccagcaggg gtcatcccagtggacacggggtcgcttccccctacccggcccttccgtctccggtccgtggacgggggtcgtccc
gcagaggctgtgggcagccgggaggctcccagaggctccgacagaatgggagtggggttgagcccacccctcact cgtctccgacacccgtcggccctccgagggtctccgaggctgtcttaccctcaccccaactcgggtggggagtga
gcagcccaggaacctgagcccagagggggccacccaccttccccaggcagggaggcccggcccccagggagatgg cgtcgggtccttggactcgggtctcccccggtgggtggaaggggtccgtccctccgggccgggggtccctctacc
gggggatgggggaggagaagggcctgcccccacccggcagcctcaggaggggcagctcgggcgggatatggaaag ccccctaccccctcctcttcccggacgggggtgggccgtcggagtcctccccgtcgagcccgccctatacctttc
aggccacagcagtgagcagagacacagaggaggaaggggccctgagctggggagacccccacggggtagggcgtg tccggtgtcgtcactcgtctctgtgtctcctccttccccgggactcgacccctctgggggtgccccatcccgcac
Red: Primers
Blue: CpG sites
13 16 20 32 36 44 45 55

## Slide 2
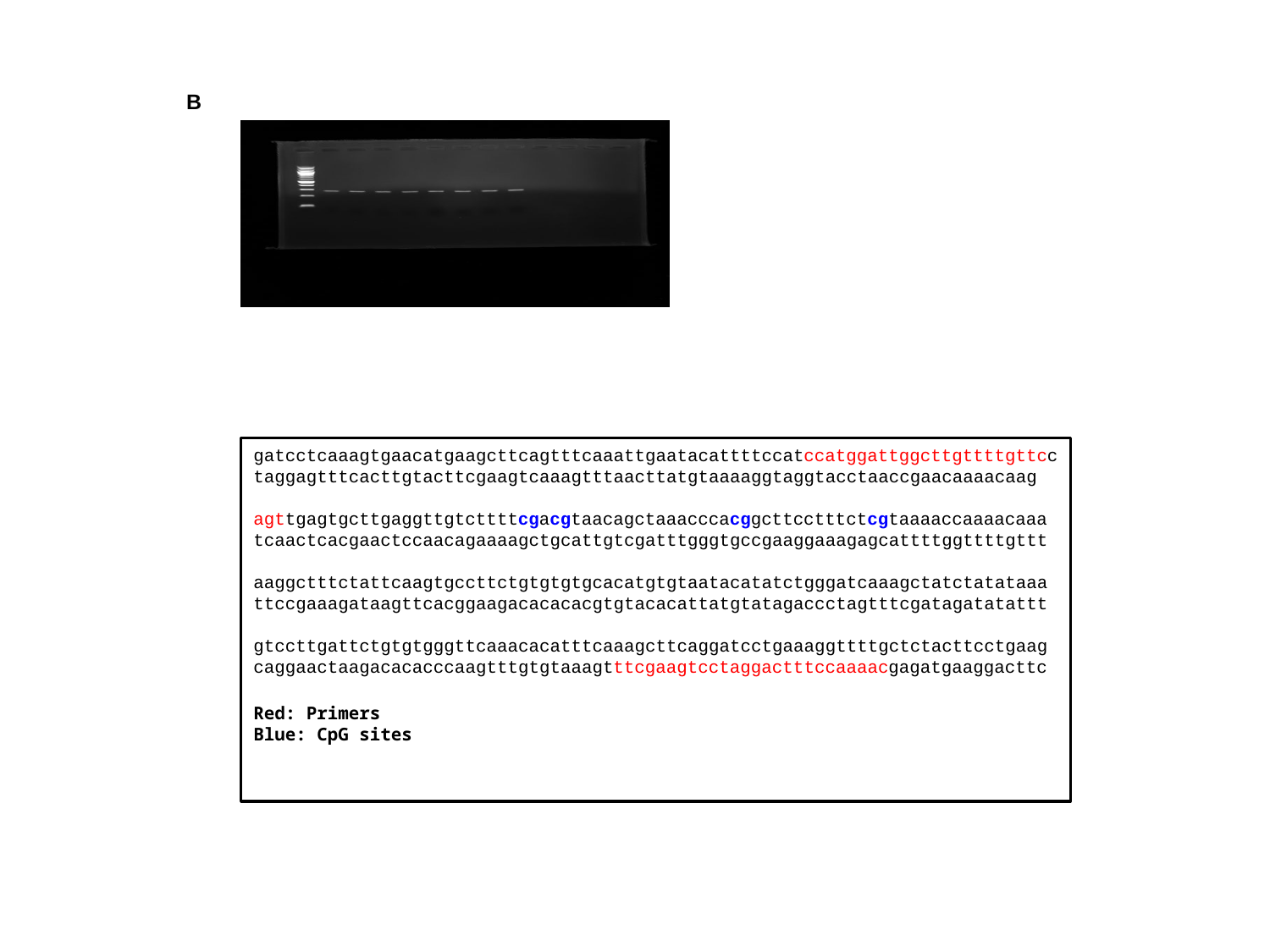

B
gatcctcaaagtgaacatgaagcttcagtttcaaattgaatacattttccatccatggattggcttgttttgttcctaggagtttcacttgtacttcgaagtcaaagtttaacttatgtaaaaggtaggtacctaaccgaacaaaacaag
agttgagtgcttgaggttgtcttttcgacgtaacagctaaacccacggcttcctttctcgtaaaaccaaaacaaa
tcaactcacgaactccaacagaaaagctgcattgtcgatttgggtgccgaaggaaagagcattttggttttgttt
aaggctttctattcaagtgccttctgtgtgtgcacatgtgtaatacatatctgggatcaaagctatctatataaa ttccgaaagataagttcacggaagacacacacgtgtacacattatgtatagaccctagtttcgatagatatattt
gtccttgattctgtgtgggttcaaacacatttcaaagcttcaggatcctgaaaggttttgctctacttcctgaag caggaactaagacacacccaagtttgtgtaaagtttcgaagtcctaggactttccaaaacgagatgaaggacttc
Red: Primers
Blue: CpG sites
13 16 20 32 36 44 45 55

## Slide 3
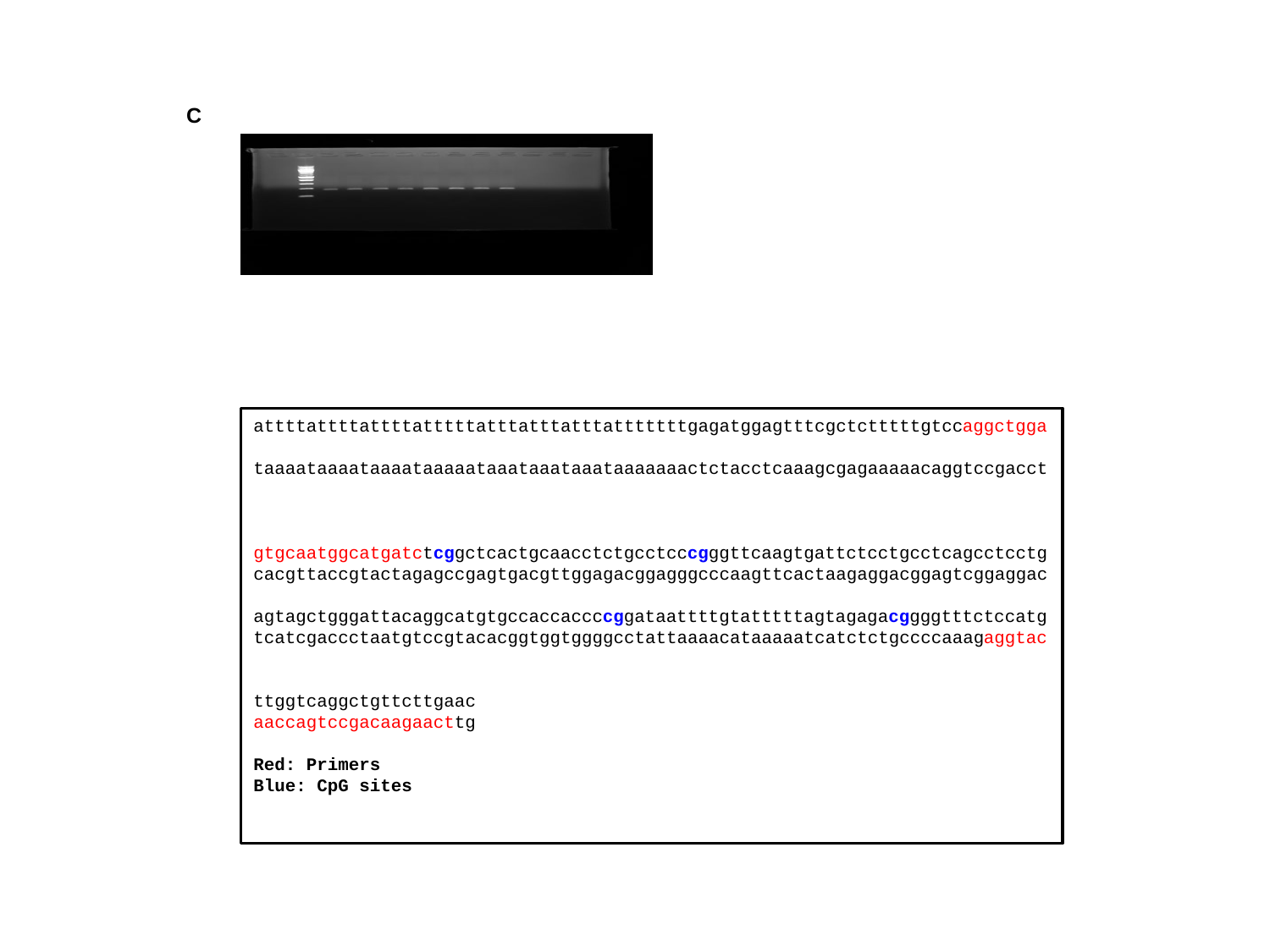

C
attttattttattttatttttatttatttatttatttttttgagatggagtttcgctctttttgtccaggctgga taaaataaaataaaataaaaataaataaataaataaaaaaactctacctcaaagcgagaaaaacaggtccgacct
gtgcaatggcatgatctcggctcactgcaacctctgcctcccgggttcaagtgattctcctgcctcagcctcctg cacgttaccgtactagagccgagtgacgttggagacggagggcccaagttcactaagaggacggagtcggaggac
agtagctgggattacaggcatgtgccaccaccccggataattttgtatttttagtagagacggggtttctccatg tcatcgaccctaatgtccgtacacggtggtggggcctattaaaacataaaaatcatctctgccccaaagaggtac
ttggtcaggctgttcttgaac
aaccagtccgacaagaacttg
Red: Primers
Blue: CpG sites
13 16 20 32 36 44 45 55

## Slide 4
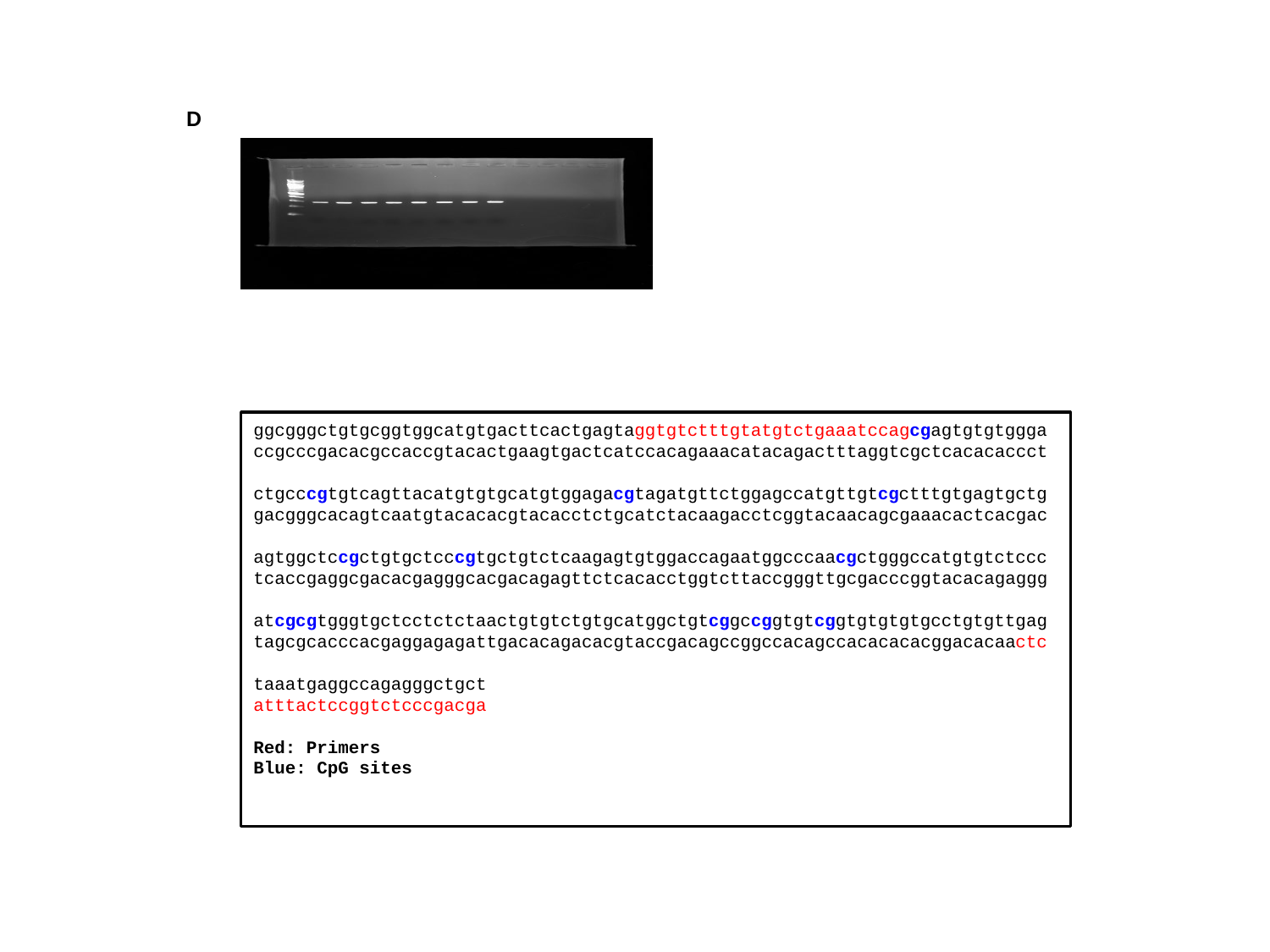

D
ggcgggctgtgcggtggcatgtgacttcactgagtaggtgtctttgtatgtctgaaatccagcgagtgtgtggga ccgcccgacacgccaccgtacactgaagtgactcatccacagaaacatacagactttaggtcgctcacacaccct
ctgcccgtgtcagttacatgtgtgcatgtggagacgtagatgttctggagccatgttgtcgctttgtgagtgctg
gacgggcacagtcaatgtacacacgtacacctctgcatctacaagacctcggtacaacagcgaaacactcacgac
agtggctccgctgtgctcccgtgctgtctcaagagtgtggaccagaatggcccaacgctgggccatgtgtctccc
tcaccgaggcgacacgagggcacgacagagttctcacacctggtcttaccgggttgcgacccggtacacagaggg
atcgcgtgggtgctcctctctaactgtgtctgtgcatggctgtcggccggtgtcggtgtgtgtgcctgtgttgag
tagcgcacccacgaggagagattgacacagacacgtaccgacagccggccacagccacacacacggacacaactc
taaatgaggccagagggctgct
atttactccggtctcccgacga
Red: Primers
Blue: CpG sites
13 16 20 32 36 44 45 55

## Slide 5
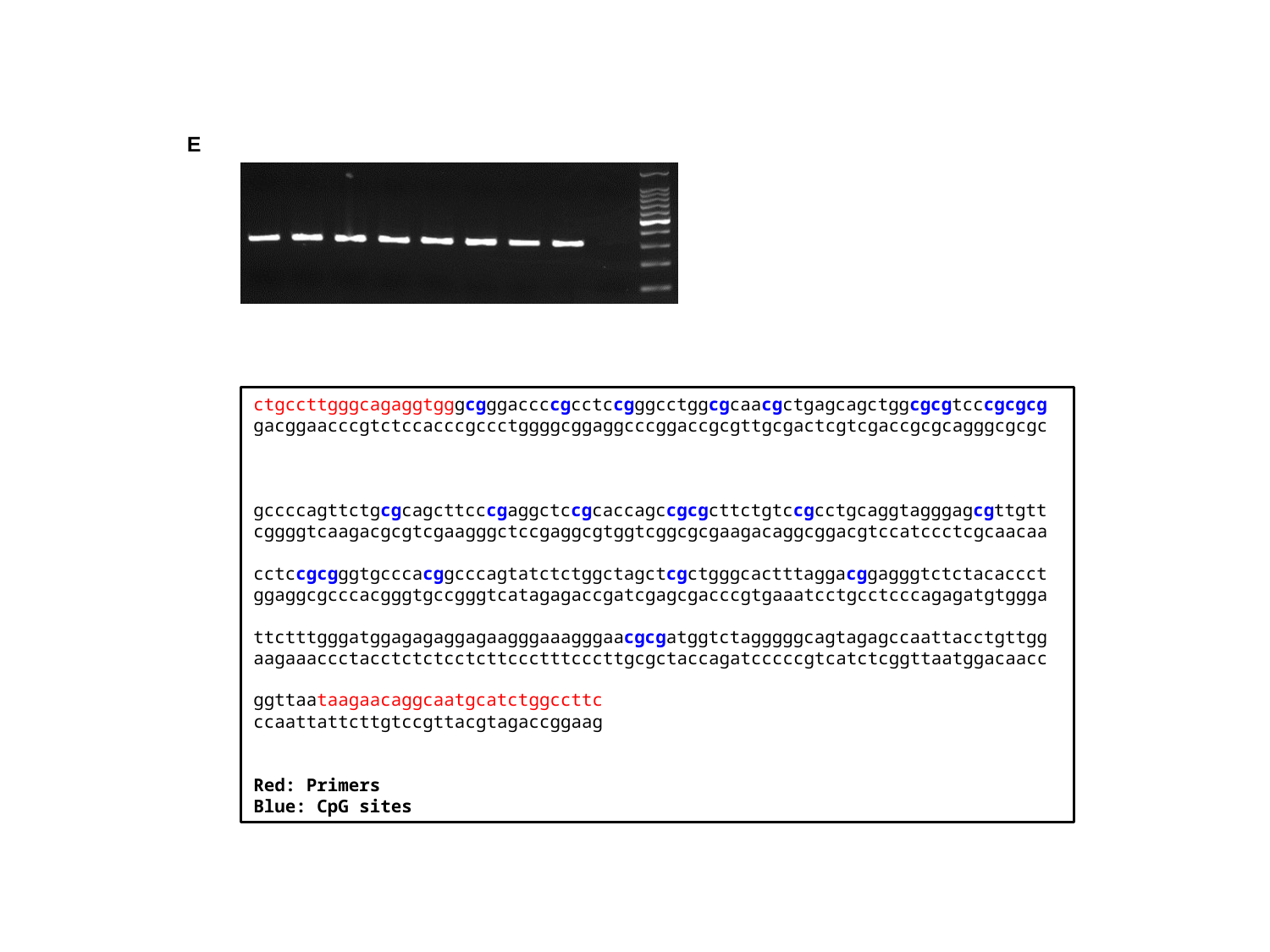

E
ctgccttgggcagaggtgggcgggaccccgcctccgggcctggcgcaacgctgagcagctggcgcgtcccgcgcg
gacggaacccgtctccacccgccctggggcggaggcccggaccgcgttgcgactcgtcgaccgcgcagggcgcgc
gccccagttctgcgcagcttcccgaggctccgcaccagccgcgcttctgtccgcctgcaggtagggagcgttgtt
cggggtcaagacgcgtcgaagggctccgaggcgtggtcggcgcgaagacaggcggacgtccatccctcgcaacaa
cctccgcgggtgcccacggcccagtatctctggctagctcgctgggcactttaggacggagggtctctacaccct
ggaggcgcccacgggtgccgggtcatagagaccgatcgagcgacccgtgaaatcctgcctcccagagatgtggga
ttctttgggatggagagaggagaagggaaagggaacgcgatggtctagggggcagtagagccaattacctgttgg aagaaaccctacctctctcctcttccctttcccttgcgctaccagatcccccgtcatctcggttaatggacaacc
ggttaataagaacaggcaatgcatctggccttc
ccaattattcttgtccgttacgtagaccggaag
Red: Primers
Blue: CpG sites
13 16 20 32 36 44 45 55

## Slide 6
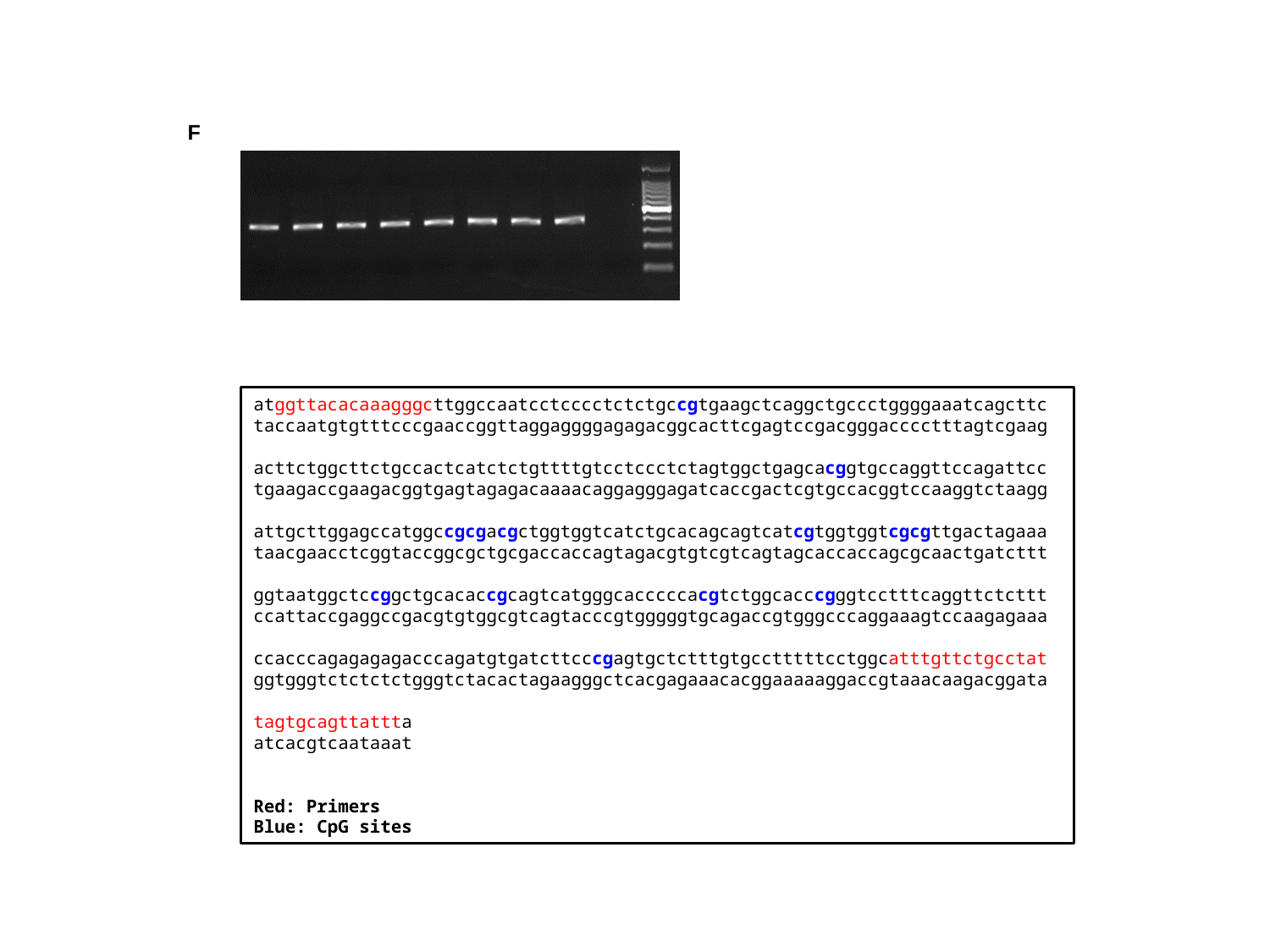

F
atggttacacaaagggcttggccaatcctcccctctctgccgtgaagctcaggctgccctggggaaatcagcttc
taccaatgtgtttcccgaaccggttaggaggggagagacggcacttcgagtccgacgggacccctttagtcgaag
acttctggcttctgccactcatctctgttttgtcctccctctagtggctgagcacggtgccaggttccagattcc
tgaagaccgaagacggtgagtagagacaaaacaggagggagatcaccgactcgtgccacggtccaaggtctaagg
attgcttggagccatggccgcgacgctggtggtcatctgcacagcagtcatcgtggtggtcgcgttgactagaaa taacgaacctcggtaccggcgctgcgaccaccagtagacgtgtcgtcagtagcaccaccagcgcaactgatcttt
ggtaatggctccggctgcacaccgcagtcatgggcacccccacgtctggcacccgggtcctttcaggttctcttt ccattaccgaggccgacgtgtggcgtcagtacccgtgggggtgcagaccgtgggcccaggaaagtccaagagaaa
ccacccagagagagacccagatgtgatcttcccgagtgctctttgtgcctttttcctggcatttgttctgcctat ggtgggtctctctctgggtctacactagaagggctcacgagaaacacggaaaaaggaccgtaaacaagacggata
tagtgcagttattta
atcacgtcaataaat
Red: Primers
Blue: CpG sites
13 16 20 32 36 44 45 55
